# Polyphosphate (PolyP) for alveolar cleft repair: study protocol for a pilot randomized controlled trial

**DOI:** 10.1186/s13063-021-05325-2

**Published:** 2021-06-14

**Authors:** S. A. Alkaabi, D. S. Natsir Kalla, G. A. Alsabri, A. Fauzi, A. Tajrin, W. E. G. Müller, H. C. Schröder, X. G. Wang, T. Forouzanfar, M. N. Helder, M. Ruslin

**Affiliations:** 1 Department of Oral and Maxillofacial Surgery/Oral Pathology, Amsterdam University Medical Centers and Academic Centre for Dentistry Amsterdam (ACTA), Amsterdam Movement Sciences, Vrije Universiteit Amsterdam, Amsterdam, The Netherlands; 2 Department of Oral and Maxillofacial Surgery, Al Kuwait Hospital, Ministry of Health, Dubai, United Arab Emirates; 3 Department of Biochemistry, Faculty of Medicine, Hasanuddin University, Makassar, Indonesia; 4 Department of Oral and Maxillofacial Surgery, Faculty of Dentistry, Hasanuddin University, Makassar, 90425 Indonesia; 5 Institut für Physiologische Chemie, Angewandte Molekularbiologie, Universitätsmedizin, Johannes Gutenberg-Universität Mainz, Mainz, Germany; 6 NanotecMARIN GmbH, Mainz, Germany

**Keywords:** Polyphosphate, Alveolar bone grafting, Bone regeneration, Regenerative medicine

## Abstract

**Objective:**

Bone grafting is an important surgical procedure to restore missing bone in patients with alveolar cleft lip/palate, aiming to stabilize either sides of the maxillary segments by inducing new bone formation, and in bilateral cleft cases also to stabilize the pre-maxilla. Polyphosphate (PolyP), a physiological polymer composed of orthophosphate units linked together with high-energy phosphate bonds, is a naturally existing compound in platelets which, when complexed with calcium as Ca-polyP microparticles (Ca-polyP MPs), was proven to have osteoinductive properties in preclinical studies.

**Aim:**

To evaluate the feasibility, safety, and osteoinductivity of Ca-polyP MPs as a bone-inducing graft material in humans.

**Methods:**

This prospective non-blinded first-in-man clinical pilot study shall consist of 8 alveolar cleft patients of 13 years or older to evaluate the feasibility and safety of Ca-PolyP MPs as a bone-inducing graft material. Patients will receive Ca-polyP graft material only or Ca-polyP in combination with biphasic calcium phosphate (BCP) as a bone substitute carrier. During the trial, the participants will be investigated closely for safety parameters using radiographic imaging, regular blood tests, and physical examinations. After 6 months, a hollow drill will be used to prepare the implantation site to obtain a biopsy. The radiographic imaging will be used for clinical evaluation; the biopsy will be processed for histological/histomorphometric evaluation of bone formation.

**Discussion:**

This is the first-in-man study evaluating the safety and feasibility of the polyP as well as the potential regenerative capacity of polyP using an alveolar cleft model.

**Trial registration:**

Indonesian Trial Registry INA-EW74C1N. Registered on 12 June 2020

## Administrative information

### Trials guidance

**Table:** 

Title	Polyphosphate (PolyP) for alveolar cleft repair, study protocol for a pilot randomized controlled trial. A total of eight patients, four patients (randomized) will receive Ca-PolyP MP as bone graft, and the other 4 patients will receive a combination of PolyP/BCP as graft material
**Trial registration**	Indonesian Trial Registry under number INA-EW74C1N. Ethical committee of Faculty of Medicine, Hasanuddin University, Makassar, Indonesia 1063/UN4.6.4.5.31/PP36/2019.
**Protocol version**	Version 1.0, dated 28 May 2019
**Funding**	No funding was received
**Author details**	1. Alkaabi SA & Natsir Kalla DS: Dept. of Oral and Maxillofacial Surgery/Oral Pathology, Amsterdam University Medical Centers and Academic Centre for Dentistry Amsterdam (ACTA), Vrije Universiteit Amsterdam, Amsterdam Movement Sciences, Amsterdam, The Netherlands. Role: Main author and Conceptualization and Writing. 2. Alsabri GA: Dept. of Oral and Maxillofacial Surgery/Oral Pathology, Amsterdam University Medical Centers and Academic Centre for Dentistry Amsterdam (ACTA), Vrije Universiteit Amsterdam, Amsterdam Movement Sciences, Amsterdam, The Netherlands. Role: Reviewer and editing. 3. Ruslin M , Fauzi A & Tajrin A: Dept. of Oral and Maxillofacial Surgery, Faculty of Dentistry, Hasanuddin University, Makassar, Indonesia. Role: Surgical procedures. 4. Ruslin M: Dept. of Oral and Maxillofacial Surgery, Faculty of Dentistry, Hasanuddin University, Makassar, Indonesia. Role: Correspondance. 5. Müller WEG, Schröder HC & Wang XG: Institut für Physiologische Chemie, Angewandte Molekularbiologie, Universitätsmedizin, Johannes Gutenberg-Universität Mainz, Mainz, Germany. Role: PolyP Inventor. 6. Forouzanfar T& Helder MN: Dept. of Oral and Maxillofacial Surgery/Oral Pathology, Amsterdam University Medical Centers and Academic Centre for Dentistry Amsterdam (ACTA), Vrije Universiteit Amsterdam, Amsterdam Movement Sciences, Amsterdam, The Netherlands. Role: Methodology and supervision.
**Name and contact information for the trial sponsor**	Muhammad Ruslin Department of Oral and Maxillofacial Surgery Faculty of Dentistry Hasanuddin University Kode Pos 90425 Makassar Indonesia Tel: +62-41-158-6012 Fax: +62-41-143-3015
**Role of sponsor**	There was no sponsor.

## Background

Alveolar cleft is a defect occurring as a result of the failure of regular development during frontonasal prominence growth, which mostly affects the site between the lateral incisor and the canine (Von Eiselsberg F., 1901). In 1901, the alveolar bone cleft defect was first reconstructed by von Eiselsberg using an autogenous bone graft, while Lexer published in 1908 the first reconstruction with nonvascular graft material [1, 2]. The autogenous bone most often derived from the cancellous iliac crest is still considered as a golden standard for the grafting procedure. Other sources such as the tibia, mandibular symphysis, rib, and the cranium are still being used by surgeon preference [3–7]. However, the drawback of autogenous graft is that it requires another surgical site, which may be associated with post-operative complications [8]. Consequently, the development of effective bone graft substitutes is currently being given high priority and attention [9, 10].

Müller and colleagues identified a new bone graft based on polyphosphate (polyP) [11, 12]. PolyP is a naturally existing compound in the platelets [13]; a physiological polymer composed of orthophosphate units linked together with high-energy phosphate bonds similar to ATP [14]. Complexed with calcium as Ca-polyP microparticles (Ca-polyP MPs), it was proven to have osteoinductive properties in preclinical studies [14–16]. PolyP is also used as a food additive (E 452) and in cosmetics [17]. As such, polyP is considered a safe material in current human applications [18].

Biphasic calcium phosphate (BCP) is a mixture of hydroxyapatite (HA) and β-tricalcium phosphate (β-TCP) with different ratios [19]. BCP in some reports showed intrinsic osteoinductive properties causing ectopic bone formation [20, 21]. While other reports such as de Lange et al. showed that BCP has osteoconductive properties facilitating the bone formation and remodeling in a maxillary sinus lift model [22].

The aim of the current phase I clinical protocol study is to test the safety and feasibility of amorphous Ca-polyP MPs as a graft material.

## Objective

The protocol of this study as presented here is first-in-human.

### Primary objective

The primary objective is to assess the safety of amorphous Ca-polyP MPs as a graft material in the human alveolar cleft reconstruction model.

### Secondary objective

The secondary objective is to evaluate the feasibility and the potential regenerative capacity of polyP using an alveolar cleft model amorphous Ca-polyP MPs.

We hypothesize that the bony reconstruction with osteoinductive Ca-polyP MPs, either or not in combination with BCP granulate, will accelerate the quantity and quality of bone formation in a timely manner. Further, it will reduce the surgical time and morbidity by the absence of a donor site, thereby increasing the cost-effectiveness and quality of care.

## Methods and design

### Ethics

The clinical trial was approved by the Ethics and Research Committee of Faculty of Medicine, Hasanuddin University, Makassar, Indonesia, with code number 1063/UN4.6.4.5.31/PP36/2019. Participants will be recruited from general practices of Hasanuddin Dental Hospital and in the area around Makassar. The trial will be conducted in Hasanuddin Dental Hospital. All participants shall be asked to sign an informed consent. This study complies with the principles of the Declaration of Helsinki.

### Study design

This is a single-center prospective control clinical trial that will be conducted in Hasanuddin University, Hasanuddin Dental Hospital, to assess the safety and feasibility of calcium-polyphosphate microparticles (Ca-polyP MPs, CAS No.: 13477-39-9, EC No.: 236-769-6) as a bone graft material in an alveolar cleft model. The average MP particle size diameter is 280 ± 120 nm [12]. A total of 8 patients will be included in the trial using a parallel assignment intervention. Four patients (randomized) will receive Ca-PolyP MP as a bone graft, and the other 4 patients will receive a combination of PolyP/BCP as a graft material. The primary endpoint will be set at 6 months. At each follow-up visit, AE and/or SAEs will be documented, and clinical assessments will be performed at time points specified in the “Intervention” section. All patients will be monitored closely using lab tests (complete blood count (10.1053/jpan.2003.50013), others if needed), radiographs, and periodic physical examination (Table 1). After these 6 months, a bone biopsy will be taken during dental implant preparation and processed for histological/histomorphometric analysis. Finally, a report on safety, feasibility, and potential efficacy with regard to bone formation will be made and will, irrespective of the outcomes, be published in a peer-reviewed journal.

**Table 1:** Assessment table {13}

	Consent form	Panorama	CBCT or CT	Physical examination	CBC	Thermometer	Biopsy
Pre-operatively	✓	✓	✓	✓	✓	✓	✓
Operative day				✓		✓	
Post-op day1		✓		✓	✓	✓	
Post-op day 8		✓	✓	✓	✓	✓	
Post-op day14				✓		✓	
Post-op day 30				✓	✓	✓	
Post-op day 90		✓		✓		✓	
Post-op day 180		✓	✓	✓	✓	✓	✓

*CT* computed tomography, *CBCT* cone beam CT, *CBC* complete blood count

### Eligibility criteria

#### Inclusion and exclusion criteria

After written informed consent will be obtained by a research team member, the participant will be screened further for eligibility. Patients should be ≥ 13 years old, healthy male or female patients with an alveolar cleft bone defect, non-smoker, with no history of previous grafting procedure(s), with a normal blood count, and with an ASA1 regarding anesthetic risks.

Patients will be excluded when they have poor oral hygiene with mouth plaque, are over 70 years old, are classified as ASA3 and beyond, have local infection and active systematic disease, or received radiotherapy, chemotherapy, immunosuppressive, or anticoagulant therapy recently. Other exclusion criteria comprise having received bone morphogenetic protein (BMP) growth factors or other bone growth-promoting factor therapy, obvious malnutrition, and active influenza.

#### Withdrawal of participants

Participants can leave the study at any time for any reason without any consequences. The investigator can decide to withdraw a subject from the study for urgent medical reasons. When participants withdraw prior to grafting intervention, they will be replaced. Furthermore, if a membrane has been used for any reason, the patient will be considered as a dropout and will be replaced.

### Intervention

Under general anesthesia, and after local infiltration with adrenaline 1:100,000, an incision will be made at the cleft margin to create a pocket-like tissue towards the nose and the mouth in order to reconstruct the nasal floor as well as the palatal tissue. The goal of this approach is to get rid of the oro-nasal fistula and to expose the bony edges on both sides of the cleft. Under sterile conditions, either Ca-polyP MP alone (NanotecMARIN GmbH, Mainz, Germany) or a combination of BCP (Straumann Bone Ceramic, Villeret, Switzerland) and PolyP will be mixed with normal saline in a ratio of 1 g:1.5 ml and 1 g:2 g:3–5 ml, respectively. A homogenous mixture should be reached before placing the graft material into the cleft defect. A good adaptation of bone graft material should be considered while placing it in the cleft defect. No membrane will be used. A different graft quantity will be considered for larger defects, however, with the same mixing ratios. Absorbable sutures with 3/0 Vicryl for the mucosa and 4/0 Vicryl for the nasal reconstruction will be used for closure.

Post-operative, suitable antibiotics and painkillers will be prescribed to all patients.

### Adverse event (AE) and serious adverse event (SAE)

Any adverse event will be graded with respect to intensity and classified as either serious or non-serious according to the World Health Organization classification. Any change in health which occurs between screening examination and first administration of amorphous Ca-polyP microparticles or related procedures will be recorded as part of the subject’s medical history, and full medical care will be given to all participants. In the case of a SAE, the sponsor will be notified within 24 h from the onset. If the SAE concerns severe toxicity or infection associated with the graft site, the trial will be terminated immediately.

### Sample size

Since this is a first-in-man trial, the current trial sample size has been limited to only 2 × 4 patients, with the primary goal to gain a first insight on the safety and feasibility of the treatment with Ca-polyP. It is assumed that no SAEs or AEs will occur, and then, an n = 4 for each group should therefore be sufficient.

### Recruitment

Prior to recruitment, an audit will be carried out by the surgical and ethical team to evaluate the safety measurements at the research site in the Hasanuddin Dental Hospital. Patients will be recruited from an existing database of patients eligible for the proposed treatment available from the Hasanuddin University, Hasanuddin Dental Hospital.

### Randomization and treatment allocation

Because this is a first-in-human study, it is not possible to keep all personnel blinded to the assignment group. After written informed consent will be obtained by the main surgeon, randomization will be performed with regard to the treatment group. Central randomization using a randomization program on a secure computer will be used after the completion of patient enrollment. Patients will receive a unique study code, and their data will be provided to the clinical and research evaluators in a patient-coded manner.

### Blinding

**Fig. 1:**
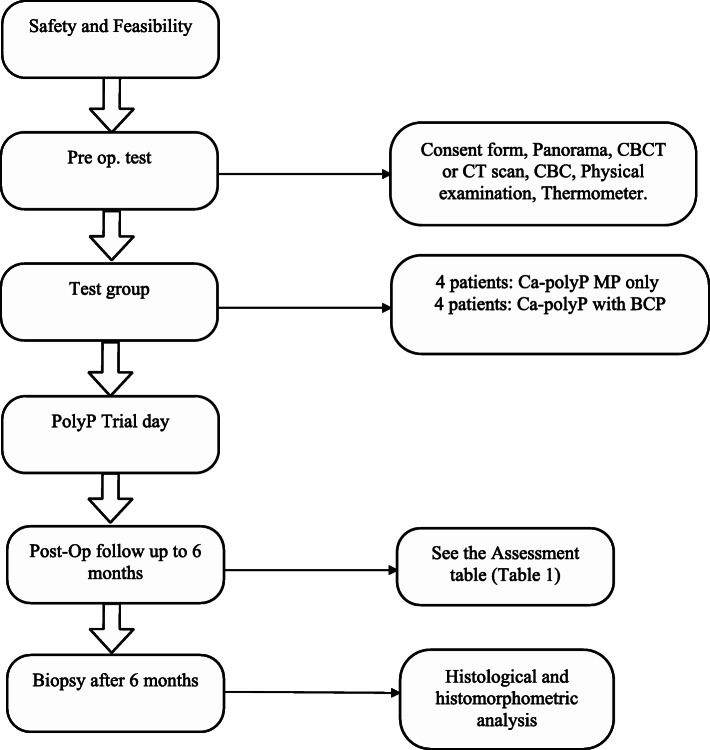
Protocol flowchart

The radiologist and the histopathologist will be kept blinded to the treatment when evaluating the data (Fig. 1).

### Data collection and access

The rules and responsibilities will be provided to the research team. The doctors and nurses of the research team will collect the data according to the evaluation (Table 1). All research team members will receive training on how to collect data at all study visits. The patient-coded data will be then handed over to the clinical evaluators and investigators. Each patient will be followed up for up to 6 months. The confidentiality of the participant’s data will be well protected by the data manager.

### Outcomes

#### Safety assessment based on physical examination and laboratory measurements

When a SAE occurs, it will be concluded that polyP is not (yet) safe in the current setting. For AEs, if they do not occur at a higher frequency than in patients treated with standard care (autologous bone) and/or can be resolved by non-invasive conventional methods (e.g., analgesics, antibiotics), the polyP product will be considered safe. In all other cases, polyP will not be considered safe (yet).

#### Radiographic evaluation

The Chelsea scale will be used to evaluate the bone graft and the level of the bone in comparison with the adjacent teeth. This scale starts with drawing an imaginary midline between the two teeth on either side of the cleft site. Each of those teeth (mesial and distal roots) will be divided starting from the cemento-enamel junction to the root apex in four parts. A 0 score is given when no bone is present up till the midline; a 0.5 score is given when there is bone, but it fails to reach the midline; and a 1 score is given when the bone extends from the root surface to the midline [23].

#### Histological and histomorphometric analysis

The histological and histomorphometric analysis will be performed in at least 3 patients from each group. In those patients, the dental implant site will be prepared using a trephine burr (⌀ 2.0 mm × 10.0 mm in length) instead of a normal drill, thereby being able to collect a biopsy from the treated site without interfering with the normal procedure. The biopsies will be fixed in 10% formalin and processed for embedding in methylmethacrylate for the evaluation of hard tissue formation. After sectioning, different stainings (Goldner’s trichrome, Toluidin blue, tartrate-resistant acid phosphatase (TRAP)) will be used, and histomorphometric parameters for bone formation will be analyzed. Two trained examiners, blinded for the treatment modality, will evaluate the images, and intra- and inter-observer reliabilities will be determined. In case of disagreement between the observers, the specimen will be re-evaluated to reach a consensus.

#### Monitoring

Monitoring will be done constantly by internal monitors of the Ethics and Research Committee of Faculty of Medicine, Hasanuddin University. Since there is a negligible risk, a data safety monitoring board will not be formed. A safety report will be provided to the Medical Research Ethics Committee of the Ethics and Research Committee of Faculty of Medicine, Hasanuddin University, every year. An interim analysis will not be conducted.

#### Statistical analysis

A SPSS power analysis for parameter comparisons between the groups will be performed. A *p* value less than 0.05 will be considered statistically significant.

#### Amendments

All substantial amendments will be notified to the ethical committee and competent authority to ensure the safety and integrity of participants as well as the scientific value of the trial.

#### Post-trial care

All participants will be kept in secondary follow-up for a period of 3 years to ensure their safety and to record any delayed side effects of the Ca-polyP graft material.

## Discussion

This is the first-in-man study evaluating the potential regenerative capacity of polyP using an alveolar cleft model. PolyP represents a completely novel type of regenerative compound, since it can be considered as a rich energy source for tissue repair, which may be as pivotal for the bone regeneration process as the osteogenic factors, which are generally believed to be the primary active compounds [14]. The high-energy phosphate bonds of polyP are identical to those present in the “common” cellular energy molecule ATP, and both serve as substrates for the enzyme alkaline phosphatase (ALP), a well-known marker for active bone formation [12]. PolyP has also been reported to promote mineralization [24] and to increase progenitor cell differentiation into osteoblasts [15, 25]. PolyP is present in platelets, which play an essential role in early wound repair. Interestingly, platelet-rich plasma (PRP), a concentrate of platelet-rich plasma protein derived from the whole blood and often used in bone repair strategies, therefore will also contain polyP. However, the efficacy of PRP to promote bone repair is nowadays questioned, since both positive and neutral/negative effects have been published recently [26, 27]. We speculate that the much higher dose of polyP present in our preparations will be well above the bone regeneration threshold, and thus may have a positive effect on the bone repair process.

Calcium phosphate ceramics including biphasic calcium phosphates (BCPs) have been widely used as bone substitutes and tissue engineering scaffolds. Calcium phosphates are highly biocompatible, proven to be safe, and successfully used in many different clinical treatment modalities such as bone augmentation in spinal arthrodesis, maxillo- and craniofacial surgeries, orthopedics, periodontal treatment, and metallic implant coatings [28–33]. Some reports describe that BCP may also have osteoinductive properties [34], which implies that BCP may add to the osteoinductivity as well. Moreover, a recent clinical study applying microstructured β-TCP for alveolar cleft repair demonstrated that calcium phosphate could be used safely and effectively for this purpose as well [35]. We are therefore convinced that the Straumann Bone Ceramic used in the current study will be a safe-to-use scaffold and may have a supportive or even synergistic effect on the bone formation when combined with the bioactive polyP.

For the clinical evaluation of bone formation, radiographic imaging will be applied. We are well aware that this will likely be relatively reliable in the case of the group that is treated only with the (radiolucent) polyP microparticles but will not be easy with the BCP/polyP treatment group. The BCP scaffold will be radiopaque and cause signal scattering, which will preclude accurate visualization of new bone formation within the scaffold material. We will circumvent this limitation by our histological and histomorphometrical analysis of the biopsies taken at the 6-month follow-up time point, during dental implant placement. This will enable us to still evaluate the bone formation at the microscopic level and to quantify multiple bone formation-related parameters and cellular activities as demonstrated before in other bone regeneration studies performed by our group [29, 30, 36, 37].

## Conclusion

With this protocol, we summarized how we intend to evaluate the safety and feasibility of Ca-polyP MP as a new grafting material in an alveolar cleft model.

## Trial status

Recruitment started in November 2019 and is planned to end in September 2020, with 8 patients randomized. The current protocol version is 1.0, dated 28 May 2019.
